# Kappa-opioid receptor stimulation in the nucleus accumbens shell and ethanol drinking: Differential effects by rostro-caudal location and level of drinking

**DOI:** 10.1038/s41386-024-01850-1

**Published:** 2024-03-25

**Authors:** Breanne E. Pirino, Annie Hawks, Brody A. Carpenter, Pelagia G. Candelas, Andrew T. Gargiulo, Genevieve R. Curtis, Anushree N. Karkhanis, Jessica R. Barson

**Affiliations:** 1 Department of Neurobiology and Anatomy, Drexel University College of Medicine, Philadelphia, PA 19129 USA; 2 Department of Psychology, Binghamton University – SUNY, Binghamton, NY 13902 USA

**Keywords:** Motivation, Molecular biology

## Abstract

Although the kappa-opioid receptor (KOR) and its endogenous ligand, dynorphin, are believed to be involved in ethanol drinking, evidence on the direction of their effects has been mixed. The nucleus accumbens (NAc) shell densely expresses KORs, but previous studies have not found KOR activation to influence ethanol drinking. Using microinjections into the NAc shell of male and female Long-Evans rats that drank under the intermittent-access procedure, we found that the KOR agonist, U50,488, had no effect on ethanol drinking when injected into the middle NAc shell, but that it promoted intake in males and high-drinking females in the caudal NAc shell and high-drinking females in the rostral shell, and decreased intake in males and low-drinking females in the rostral shell. Conversely, injection of the KOR antagonist, nor-binaltorphimine, stimulated ethanol drinking in low-drinking females when injected into the rostral NAc shell and decreased drinking in high-drinking females when injected into the caudal NAc shell. These effects of KOR activity were substance-specific, as U50,488 did not affect sucrose intake. Using quantitative real-time PCR, we found that baseline gene expression of the KOR was higher in the rostral compared to caudal NAc shell, but that this was upregulated in the rostral shell with a history of ethanol drinking. Our findings have important clinical implications, demonstrating that KOR stimulation in the NAc shell can affect ethanol drinking, but that this depends on NAc subregion, subject sex, and ethanol intake level, and suggesting that this may be due to differences in KOR expression.

## Introduction

The kappa-opioid receptor (KOR) and its endogenous neuropeptide ligand, dynorphin, are canonically associated with negative affective states [1, 2] and are implicated in both affective disorders [3–5] and substance use disorders [6], particularly alcohol use disorder [7, 8]. This KOR/dynorphin system has been shown to be altered in response to substantial ethanol experience in both humans [9] and rodents [10–14], and these changes are believed to contribute to the transition from ethanol drinking for positive reinforcement to ethanol drinking for negative reinforcement, as observed in ethanol dependence [15, 16]. Although the KOR/dynorphin system has been of significant interest in the treatment of alcohol use disorder, findings on the direction of its effects and regulation have been mixed.

A major obstacle in the treatment of alcohol use disorder is the heterogeneity in its presentation, leading to substantial individual differences in the response to treatment. Clinical studies indicate that the specific phenotype of ethanol drinking may be an important predictor of the most effective pharmacotherapy [17, 18]. Similarly, preclinical studies highlight that the level of ethanol exposure in animals can affect their response to treatment. Indeed, systemic injection with a KOR antagonist has been found to suppress high-level but not low-level ethanol drinking [19–23]. Notably, using a 20% ethanol intermittent-access procedure, we have recently found that most male rats engage in a lower-level ethanol drinking phenotype, while females can be either higher- or lower-level ethanol drinkers [24]. Thus, it may be important when investigating the KOR system to analyze its effects on ethanol drinking both by drinking level and subject sex.

The nucleus accumbens (NAc) shell is a limbic brain region with high levels of KOR expression [25], and it is especially implicated in motivated behaviors [26]. Surprisingly, despite findings of effects on drinking with a KOR antagonist [27], thus far no study has found a KOR agonist in the medial NAc shell to affect ethanol drinking [28, 29]. Importantly, KOR activation in the medial NAc shell has been shown to have opposing effects on affective behaviors, depending on the specific subregion targeted [30–32]. We have previously found that activation of the KOR in the caudal NAc shell promotes anxiety-like or avoidance behavior, but in the rostral NAc shell instead promotes approach behavior [32]. Thus, the lack of effect of KOR stimulation on ethanol drinking may be explained by the rostro-caudal location targeted, as studies have thus far targeted the middle third of the NAc shell, which we define as the middle subregion. While the available evidence suggests that KOR activity in the middle NAc shell does not alter ethanol drinking, it remains to be determined if the opposing effects on affective behavior of the KOR in the rostral and caudal NAc shell extend to ethanol drinking.

In this study, we examined if the KOR in subregions of the NAc shell has differential effects on ethanol intake, depending on the phenotype of the ethanol drinker, and if this could occur due to differences in expression of the KOR. Our hypothesis was that KOR activity in the NAc shell can differentially affect ethanol drinking, depending on the prior level of ethanol intake, sex of the drinker, and specific NAc shell subregion targeted, and that differences in the response to KOR stimulation may be explained by differences in KOR expression.

## Materials and Methods

Also see Supplementary Materials and Methods.

### Subjects

Male and female Long-Evans rats (*N* = 160; 59 males and 101 females, 7 weeks old at the start of ethanol access, Charles River Laboratories, Inc., Malvern, PA, USA) were individually housed in an AAALAC-accredited facility on a 12-h reversed light/dark cycle (lights off at 0900 h). Rats were handled prior to experiments and were given at least one week to acclimate to the facility. They received ad libitum access to water and chow (Laboratory Rodent Diet 5001, Lab Diet, St. Louis, MO, USA). Experiments were approved by the Institutional Animal Care and Use Committee of Drexel University College of Medicine and followed the NIH Guide for the Care and Use of Laboratory Animals.

### Experimental protocol

#### Experiment 1

To determine the effects of KOR stimulation in subregions of the NAc shell on ethanol drinking, male and female rats (*N* = 73; *n* = 8–9/group) were given access to 20% v/v ethanol in an intermittent-access two-bottle-choice procedure, bilaterally cannulated after 5 weeks of drinking for injections into the rostral, middle, or caudal NAc shell, and injected (during drinking weeks 8–9) 10 min prior to ethanol access with the selective KOR agonist (±)-trans-U50,488 hydrochloride (U50,488) counterbalanced against saline vehicle in a within-subject Latin-square design across ethanol access days. Following injection, intake of ethanol, food, and water was measured at 30 min, the period during which animals drink ethanol in a binge-like manner [24], and at the end of their daily, 24-hour access.

#### Experiment 2

To determine if the effects of KOR stimulation are specific to ethanol, male and female rats (*N* = 32; *n* = 7–9/group) were given access to 2.5% w/v sucrose in an intermittent-access two-bottle choice procedure, cannulated after 5 weeks of drinking for injections into the rostral or caudal NAc shell, and injected (during drinking weeks 8–9) 10 min prior to sucrose access with U50,488 counterbalanced against saline vehicle in a within-subject Latin-square design across sucrose access days. Since we found in Experiment 1 that the effect of U50,488 produced opposing effects on ethanol drinking when injected into the rostral vs. caudal NAc shell of male rats but instead produced opposing effects in low vs. high drinkers when injected into the rostral shell of females, cannulas were implanted into the rostral and caudal NAc shell in males (all low drinkers) but in the rostral shell of high- and low-drinking females. Due to poor health, 3 low-drinking females were euthanized prior to injections. Following injection, intake of sucrose, food, and water was measured at 30 min and 24 h into access.

#### Experiment 3

To determine if endogenous KOR stimulation in the rostral and caudal subregions of the NAc shell could affect ethanol drinking, female rats from Experiment 1 and additional male and female rats treated identically except without prior U50,488 (*N* = 51; *n* = 8/group), were injected (during weeks 9–10) 22 h prior to ethanol access with nor-binaltorphimine dihydrochloride (nor-BNI) [2, 27] or saline vehicle in a within-subject design. Two male rats were removed from the study due to poor health prior to microinjections. Because nor-BNI is a long-acting KOR antagonist [33], saline injections always occurred first [27]. The delay between injections and ethanol access was to account for evidence that nor-BNI may first act as a mu-opioid receptor antagonist [34, 35]. Intake of ethanol, food, and water was measured at 30 min and 24 h into access.

#### Experiment 4

To determine if ethanol drinking alters KOR and, for comparison, dynorphin gene expression in the rostral and caudal NAc shell, female rats (*N* = 17; *n* = 8–9/group) were given access to 20% ethanol in an intermittent-access two-bottle choice procedure or water and chow only. Because females overall showed higher drinking levels than males and a greater range in levels, we examined gene expression only in female rats. They were sacrificed after 10 weeks, at the time they would normally receive access to ethanol but without ethanol on board, for quantitative real-time PCR to measure levels of KOR (Oprk1) and dynorphin (prodynorphin) mRNA.

### Statistical analysis

Statistical analysis was conducted using IBM SPSS Statistics version 28 (IBM, Armonk, NY, USA). Sphericity was determined using Mauchly’s test, and a Greenhouse-Geisser correction was used when sphericity was violated. Tukey’s method was used to identify statistical outliers, which were removed from analyses. To identify subgroups of ethanol drinkers, based on their average ethanol drinking, a two-step cluster analysis was used to identify the number of clusters and was then followed up with a *k*-means cluster analysis to identify which values fell into which cluster. Average daily ethanol and sucrose intake (g/kg and ml), preference, and kcal, as well as blood ethanol concentrations (BECs), were each analyzed using unpaired two-tailed *t*-tests to compare drinking phenotypes. Data collected after injection with U50,488 (Experiments 1–2) were analyzed using a mixed ANOVA for each time point, with sex, subregion, and drinking phenotype as between-subject factors and drug treatment as a within-subject factor. Because there was a main effect of sex in Experiment 1, follow-up mixed ANOVAs were conducted separately for each sex. Due to the findings in Experiment 1 suggesting that KOR effects on ethanol drinking are dependent on drinking phenotype, subject sex, and NAc shell subregion, analyses of data from Experiment 3 were performed separately on each sex and subregion, and an a priori decision was made to examine the interaction between drug treatment and drinking phenotype, regardless of significance. For females, data collected after injection with nor-BNI or vehicle (Experiment 3) were analyzed using a mixed ANOVA for each timepoint and subregion, with drinking phenotype as a between-subject factor and drug treatment as a within-subject factor. For males, intake data from Experiment 3 were analyzed using two-tailed dependent samples *t*-tests for each subregion and timepoint. Significant effects from ANOVAs were followed up by Sidak pairwise *post-hoc* comparisons. Data collected from quantitative real-time PCR (Experiment 4) were analyzed using two-tailed dependent or independent samples *t*-tests, as appropriate. All data are reported as mean ± standard error of the mean. Significance was set at *p* < 0.05. Detailed statistical results for each analysis are listed in Table S1.

## Results

### Ethanol & sucrose drinking

**Fig. 1:**
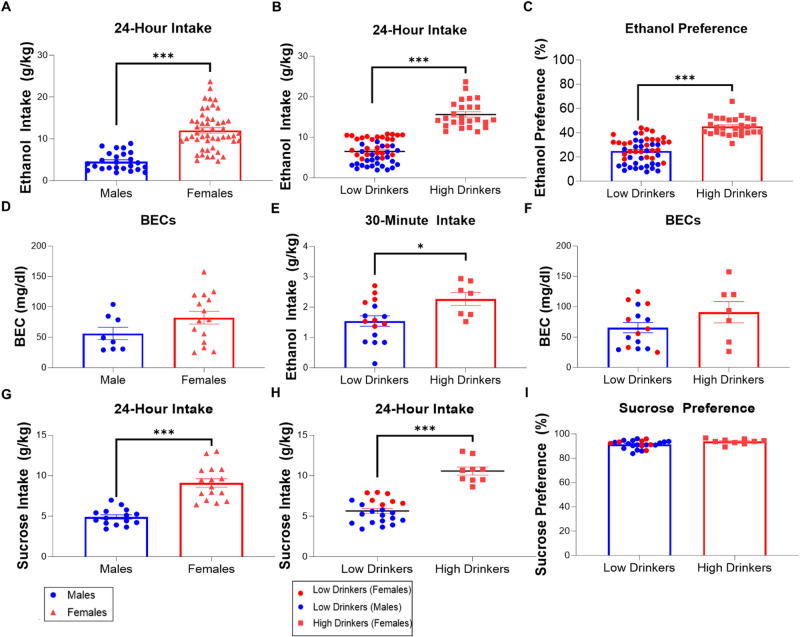
Ethanol and sucrose intake and preference. **A** For average 24-hour ethanol intake, female rats, which consumed an average of 12.00 ± 0.65 g/kg/day, drank significantly more ethanol than male rats, which consumed an average of 4.57 ± 0.43 g/kg/day. **B** A two-step cluster analysis of average 24-hour ethanol intake revealed that there were two phenotypes of ethanol drinkers, and a *k*-means cluster analysis identified that the first group consisted of all male rats (*n* = 25) and approximately half of the females (*n* = 28), which drank 6.70 ± 0.42 g/kg/day of ethanol, while the second group consisted of the other half of the female rats (*n* = 24), which drank significantly higher levels of ethanol (15.72 ± 0.69 g/kg/day) than the low drinkers. **C** High drinkers had a significantly higher preference for ethanol than low drinkers (45.12 ± 1.34 vs. 26.98 ± 1.43%). **D** For blood-ethanol concentration (BEC), male (*n* = 8) and female (*n* = 15) rats did not show a significant difference (56.26 ± 10.23 mg/dl for males vs. 82.27 ± 10.56 mg/dl for females). **E** Despite high drinkers drinking significantly more ethanol in 30 min than low drinkers (2.27 ± 0.22 g/kg/30 min vs. 1.54 ± 0.17 g/kg/30 min), **F** the difference in BECs between the high and low drinkers was not significant (90.87 ± 17.54 vs. 65.50 ± 8.36). **G** For average 24-hour sucrose intake, female rats (*n* = 16), which consumed an average of 9.10 ± 0.52 g/kg/day, drank significantly more sucrose than male rats (*n* = 16), which consumed an average of 6.21 ± 0.35 g/kg/day. **H** A two-step cluster analysis of average sucrose intake revealed that there were two phenotypes of sucrose drinkers, and a *k*-means cluster analysis identified that the first group consisted of all male rats (*n* = 16) and approximately half of the females (*n* = 7), which drank 6.51 ± 0.27 g/kg/day of sucrose, while the second group consisted of the other half of female rats (*n* = 9), which drank significantly higher levels of sucrose (10.58 ± 0.50 g/kg/day) than the low drinkers. **I** High drinkers demonstrated a trend for greater preference for sucrose than low drinkers (93.77 ± 0.73 vs. 91.41 ± 0.69%). *p < 0.05 and ***p ≤ 0.001, two-tailed independent samples *t*-tests.

Male and female rats (*N* = 109, 41 males, 68 females) were given access to ethanol (Experiments 1, 3, & 4) or sucrose (Experiment 2) under the intermittent-access two-bottle choice procedure, and their daily intake was measured for 7 weeks. Females drank significantly higher levels of ethanol than males over 24 h (*p* < 0.001) (Fig. 1A), but their BECs were not significantly different (*p* = 0.127) (Fig. 1D), despite correlating positively with ethanol intake for both females (*r* = 0.884, *p* < 0.001) and males (*r* = 0.638, *p* = 0.047). Cluster analysis of average 24-hour ethanol intake revealed that there were two phenotypes of ethanol drinkers. The first group consisted of all male rats and approximately half of the females, while the second group consisted of the other half of the females, which drank significantly higher levels of ethanol (*p* < 0.001) (Fig. 1B) and demonstrated a significantly greater ethanol preference than the low drinkers (*p* < 0.001) (Fig. 1C). Although high drinkers consumed significantly more ethanol in 30 min than low drinkers (*p* = 0.024) (Fig. 1E), the difference in BECs taken 30–40 min into daily ethanol access did not reach significance (*p* = 0.151) (Fig. 1F), despite BECs correlating positively with ethanol intake for both low (*r* = 0.927, *p* < 0.001) and high (r = 0.848, *p* = 0.016) drinkers. As with ethanol drinkers, females drank significantly more sucrose than males (*p* < 0.001) (Fig. 1G), which was also driven by a subset of high-drinking females (*p* < 0.001) (Fig. 1H); however, with high levels of sucrose preference in all rats, high sucrose-drinking rats demonstrated only a trend for greater preference for sucrose than their low-drinking counterparts (*p* = 0.059) (Fig. 1I).

### Experiment 1: KOR stimulation in the NAc shell has subregion-, sex-, and phenotype-dependent effects on ethanol drinking

**Fig. 2:**
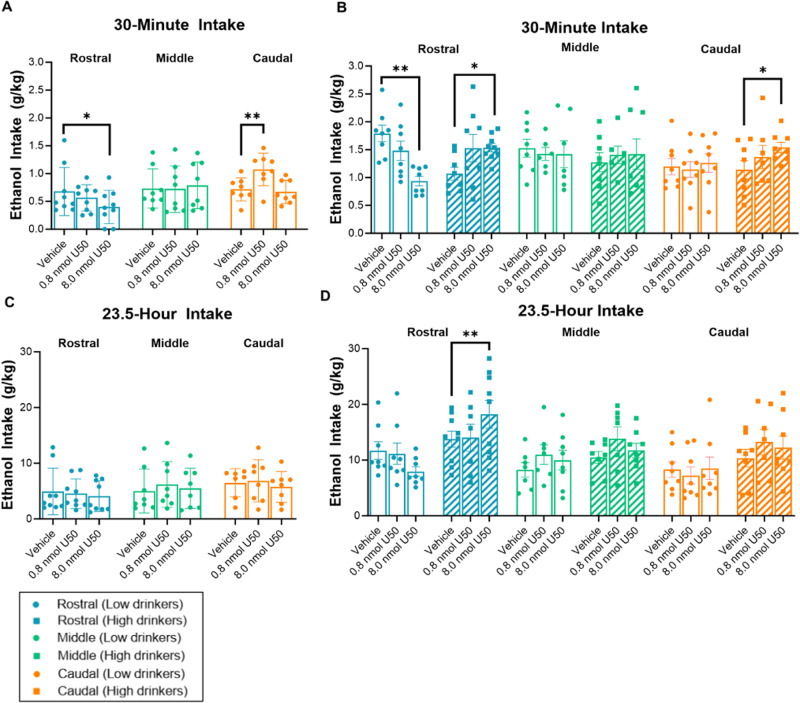
Effects of KOR agonism in subregions of the NAc shell on ethanol drinking. Effects during the first 30 min of ethanol access. **A** For male rats, although there were no significant effects of U50,488 (U50) in the *middle* NAc shell on ethanol drinking, 8.0 nmol U50 compared to saline vehicle in the *rostral* NAc shell significantly decreased ethanol drinking but 0.8 nmol U50 compared to saline vehicle in the *caudal* NAc shell significantly increased ethanol drinking. **B** For female rats, although there were no significant effects of U50 in the *middle* NAc shell on ethanol drinking, 8.0 nmol U50 compared to saline vehicle injected into the rostral NAc shell significantly decreased ethanol drinking in low drinkers but significantly increased ethanol drinking in high drinkers, and the same dose of U50 also significantly increased ethanol drinking when injected into the *caudal* NAc shell of high drinkers. **C**, **D** Effects during the remaining 23.5 h of ethanol access. **C** There were no significant effects of U50 on ethanol drinking in male rats. **D** In high-drinking rats, all of which are female, 8.0 nmol U50 compared to saline vehicle injected into the *rostral* NAc shell significantly increased ethanol drinking. *p < 0.05, **p < 0.01, and ***p ≤ 0.001 vs. vehicle, mixed ANOVAs followed-up by Sidak pairwise comparisons.

To determine the effects of KOR stimulation in subregions of the NAc shell on ethanol drinking, male and female ethanol-drinking rats (*N* = 73; *n* = 8–9/group) were bilaterally injected in the rostral, middle, or caudal NAc shell with U50,488 compared to saline vehicle. In male rats, for ethanol intake during the first 30 min of ethanol access, there was a significant main effect of drug treatment [*F*(2, 44) = 4.163, *p* = 0.022] and a significant interaction between drug treatment and subregion [*F*(4, 44) = 4.802, *p* = 0.003]. Although the KOR agonist had no effect on ethanol drinking when injected into the middle NAc shell, it promoted ethanol drinking when injected into the caudal NAc shell (*p* = 0.002) and suppressed it when injected into the rostral NAc shell (*p* = 0.029) (Figs. 2A and S4A). In females, for ethanol intake during the first 30 min of ethanol access, there was a significant main effect of drinking phenotype [*F*(1, 33) = 4.572, *p* = 0.040] and a significant interaction between drug treatment, subregion, and drinking phenotype [*F*(4, 66) = 3.203, *p* = 0.018]. As in males, while the KOR agonist had no effect on ethanol drinking when injected into the middle NAc shell, it promoted drinking when injected into the caudal NAc shell of high-drinking females (*p* = 0.038) and suppressed drinking when injected into the rostral NAc shell of low-drinking females (*p* < 0.001); however, in high-drinking females, the KOR agonist also promoted drinking when injected into the rostral NAc shell (*p* = 0.035) (Fig. 2B and S4B). Moreover, while KOR stimulation did not affect ethanol drinking after the first 30 min of access in low drinkers (males or females), KOR agonism in the rostral NAc shell of high drinkers (all females) resulted in an increase in ethanol drinking that was detectable at 24 h into daily ethanol access (*p* < 0.001) (Fig. 2C, D and S4C, D).

### Experiment 2: KOR stimulation in the NAc shell does not affect sucrose drinking

**Fig. 3:**
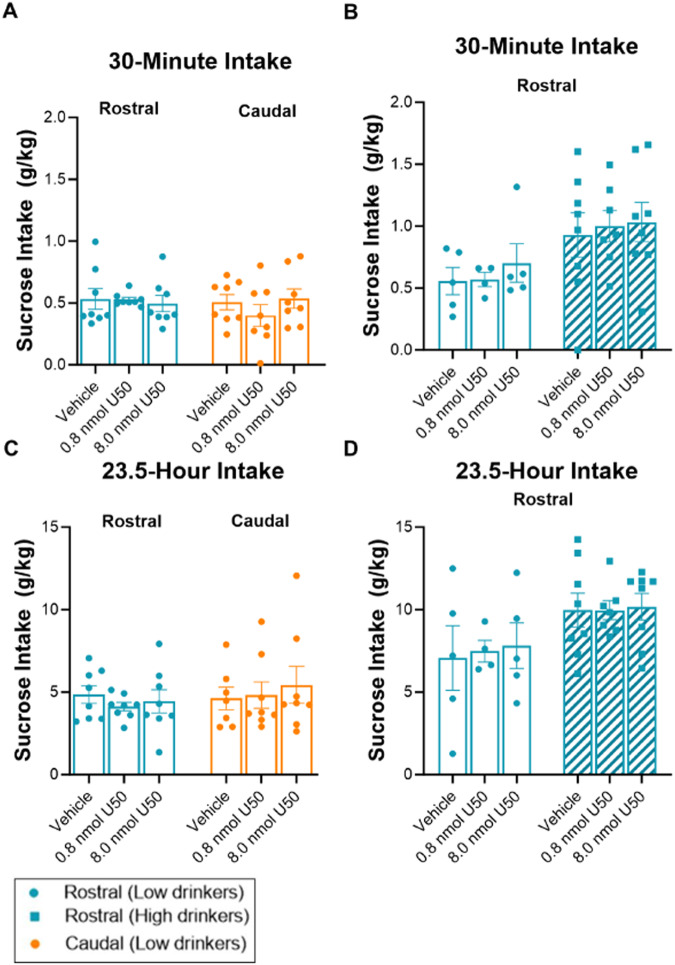
Effects of KOR agonism in subregions of the NAc shell on sucrose drinking. Effects during the first 30 min of sucrose access. **A** In male rats, there were no significant effects of U50,488 (U50) on sucrose drinking. **B** In female rats, there were no significant effects of U50 on sucrose drinking. **C**, **D** Effects during the remaining 23.5 h of sucrose access. **C** In male rats, there were no significant effects of U50 on sucrose drinking. **D** In female rats, there were no significant effects of U50 on sucrose drinking. *p < 0.05, **p < 0.01, and ***p ≤ 0 .001 vs. vehicle, mixed ANOVAs followed-up by Sidak pairwise comparisons.

To determine if the effects of KOR stimulation in subregions of the NAc shell are specific to ethanol, male and female sucrose-drinking rats (*N* = 29; *n* = 5–8/group) were injected in the rostral or caudal NAc shell with U50,488 compared to saline vehicle. Although there were differences in 30-minute sucrose drinking across drinking phenotypes [*F*(1, 24) = 17.747, *p* < 0.001], with high drinkers consuming more sucrose than low drinkers, and there were sex-related differences [*F*(1, 22) = 14.802, *p* < 0.001], with females drinking more sucrose than males after the first 30 min of access, the effects of KOR activity in the NAc shell did not extend to this other reinforcer, sucrose (Fig. 3A–D and S5).

### Experiment 3: KOR blockade in the NAc shell has subregion-, sex-, and phenotype-dependent effects on ethanol drinking

**Fig. 4:**
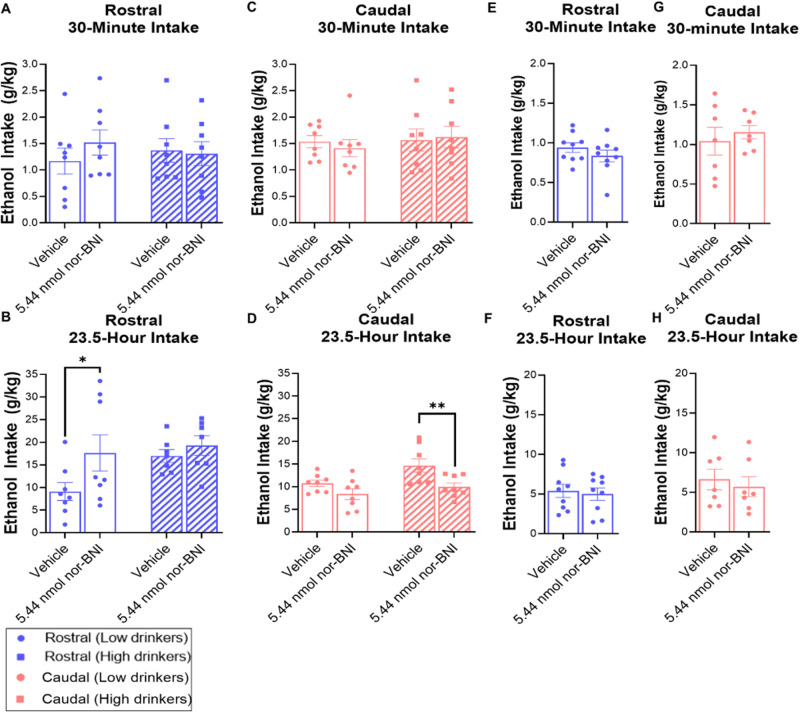
Effects of KOR antagonism in subregions of the NAc shell on ethanol drinking. In female rats, **A** there was no significant effect of KOR blockade with nor-binaltorphimine dihydrochloride (nor-BNI) on ethanol drinking via the *rostral* NAc shell during the first 30 min of ethanol intake, but **B** KOR blockade promoted ethanol drinking in low drinkers via the *rostral* NAc shell during the remaining 23.5 h of ethanol intake. **C** There was also no significant effect of KOR blockade on ethanol drinking via the *caudal* NAc shell during the first 30 min of ethanol intake, but **D** KOR blockade inhibited ethanol drinking in high drinkers via the *caudal* NAc shell during the remaining 23.5 h of ethanol intake. In male rats, **E** there was no significant effect of KOR blockade on ethanol intake via the rostral NAc shell during the first 30 min of ethanol intake or **F** during the remaining 23.5 h of ethanol intake. **G** There was also no significant effect of KOR blockade on ethanol drinking via the caudal NAc shell during the first 30 min of ethanol intake or **H** during the remaining 23.5 h of ethanol intake. *p < 0.05 and **p < 0.01 vs. vehicle.

To determine if endogenous KOR activation in subregions of the NAc shell affects ethanol drinking, male and female ethanol-drinking rats (*N* = 48, *n* = 7–9/group) were injected in the rostral or caudal NAc shell with nor-BNI compared to saline vehicle. During the first 30 min of daily ethanol access, blockade of endogenous KOR activity did not significantly alter ethanol intake in either subregion or sex (Figs. 4A, C, E, and G). During the remaining 23.5 h of ethanol access, in females injected in the rostral NAc shell, there was a significant main effect of drug treatment [*F*(1, 15) = 9.094, *p* = 0.009]. Nor-BNI compared to saline vehicle significantly increased ethanol drinking via the rostral NAc shell (*p* = 0.009), but this difference was driven by low drinkers (*p* = 0.029), with no significant difference in high drinkers (*p* = 0.085) (Figs. 4B and S6B). In females injected in the caudal NAc shell, there was also a significant main effect of drug [*F*(1,14) = 10.900, *p* = 0.005]. This time, nor-BNI compared to saline vehicle significantly decreased ethanol drinking (*p* = 0.005), and the difference was driven by high drinkers (*p* = 0.008), with no significant difference in low drinkers (*p* = 0.138) (Figs. 4D and S6D). For males, KOR blockade in neither the rostral nor the caudal NAc shell significantly affected ethanol drinking (Figs. 4F, H, and S6E–H).

### Experiment 4: KOR expression in the rostral shell is higher than in the caudal NAc shell and upregulated by ethanol experience

**Fig. 5:**
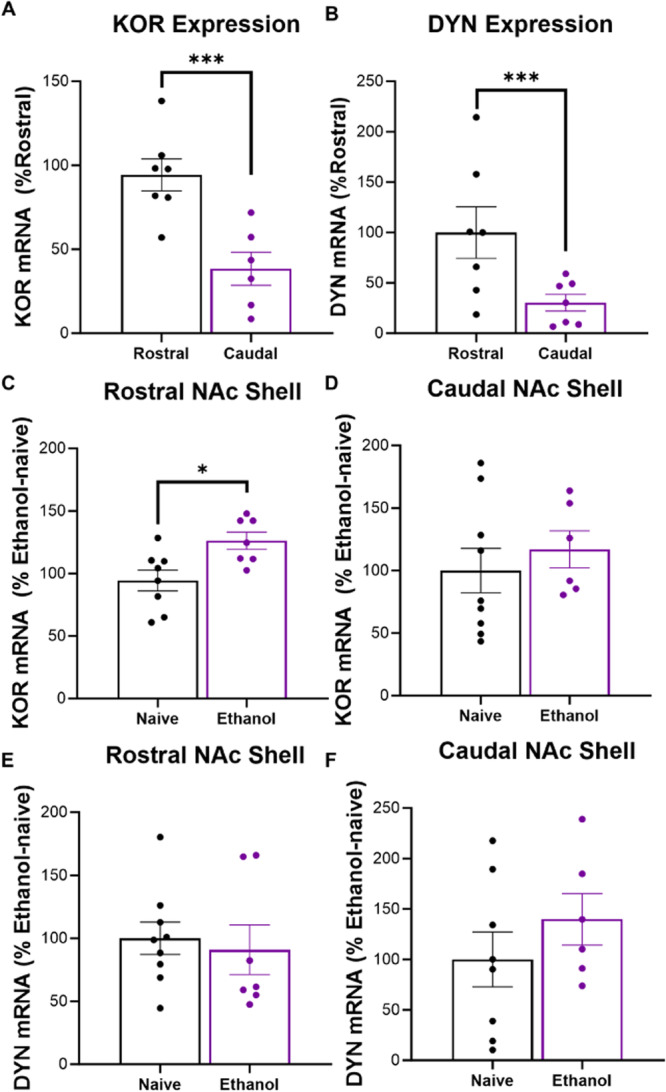
Baseline and ethanol-associated differences in KOR and dynorphin mRNA in subregions of the NAc shell. **A**, **B** Baseline comparisons of KOR and dynorphin (DYN) mRNA levels in the rostral compared to caudal NAc shell of ethanol-naïve, female rats. **A** KOR gene expression was significantly higher in the rostral compared to caudal NAc. **B** DYN gene expression was significantly higher in the rostral compared to caudal NAc shell. **C–F** Comparisons of KOR and DYN mRNA levels between ethanol-naïve rats and those with a history of ethanol drinking. **C** Ethanol history resulted in significantly increased KOR gene expression in the *rostral* NAc shell. **D** Ethanol history did not significantly affect KOR gene expression in the *caudal* NAc shell. **E** Ethanol history did not significantly affect DYN mRNA expression in the *rostral* shell. **F** Ethanol history did not significantly affect DYN mRNA expression in the *caudal* NAc shell. *p < 0.05 and ***p < 0.001, two-tailed dependent and independent samples *t*-tests.

To determine if a history of ethanol drinking affects KOR and dynorphin gene expression, real-time quantitative PCR was used to measure mRNA from the rostral and caudal NAc shell of female rats with a history of ethanol drinking and those left ethanol-naïve (*N* = 17, *n* = 6–9/group). In ethanol-naïve animals, both KOR and dynorphin were more highly expressed in the rostral compared to caudal NAc shell (*p* < 0.001) (Fig. 5A, B), but KORs were selectively upregulated in the rostral shell of those with a history of ethanol drinking (*p* = 0.012) (Fig. 5C–F).

## Discussion

Here, we demonstrate that the direction of the response to KOR stimulation in the NAc shell depends on the rostro-caudal subregion targeted and the sex and drinking phenotype of the drinker. Furthermore, we show that these differences may occur at least in part due to baseline and ethanol-associated changes in expression of the KOR.

### Sex-related drinking phenotypes

First, we found that rats demonstrate two, sex-related phenotypes of drinking, in that female rats on average drank higher levels of ethanol than male rats, but that this was driven by a subset of high-drinking females. That is, consistent with our recent findings [24], a cluster of all male rats and approximately half of the females had a lower daily ethanol intake and preference than another cluster of female rats which had a higher ethanol intake and preference. We also found that these different drinking phenotypes extended to another reinforcing substance, sucrose. In contrast with ethanol drinkers, but consistent with a similar study of Wistar rats drinking 2% sucrose [36], high and low sucrose drinkers demonstrated a similar, high preference for sucrose, perhaps reflecting a ceiling effect for their fluid intake. Thus, we recapitulated our recent finding that rats engage in two distinct and sex-related phenotypes of ethanol drinking, resulting in BECs that are not significantly different, and we demonstrated that these drinking phenotypes also occur for sucrose drinking.

### Effects of the KOR on ethanol drinking

A major finding was that the effect of KOR stimulation on ethanol drinking was dependent on the sex-related phenotype of the drinker and the rostro-caudal location targeted. Looking at male rats, previous investigations of KOR activation in the NAc shell have found no effects on ethanol drinking [28, 29], but this may be resolved by considering the rostro-caudal location targeted. Uhari-Vaananen and colleagues [29] found no effect after targeting the medial NAc shell 1.7 mm anterior to Bregma, which we define as part of the middle subregion. Similarly, Barson and colleagues [28] reported no effect after targeting 1.2 mm anterior to Bregma, which is the most rostral point of what we define as the caudal subregion, suggesting that some of their injections may have been in the middle subregion and thus is consistent with our finding that KOR agonism in the middle shell had no effect on ethanol drinking in both male and female rats. Notably, we also found that, in male rats, all of which were low drinkers, KOR agonism in the caudal NAc shell promoted drinking and KOR agonism in the rostral NAc shell suppressed drinking. Moreover, these effects were substance-specific, as there were no major changes in sucrose, water, or food intake following KOR agonism in any NAc shell subregion (see Supplementary Information). On the other hand, we found no significant effects of KOR antagonism on ethanol drinking in either the rostral or caudal subregions of male rats, consistent with previous findings of KOR blockade in the rostral NAc shell of non-dependent male rats [27]. This suggests that KOR activity may be sufficient to alter ethanol drinking in a subregion-specific manner, but it may not be necessary for ethanol drinking in non-dependent male rats. Our findings are consistent with prior literature in male rats that suggests that KOR stimulation in subregions of the medial NAc shell can lead to different and even opposite effects on affective behavior [31, 32], and they extend them to opposite effects on motivated behavior.

In female rats, the effect of KOR agonism in subregions of the NAc shell on ethanol drinking depended on the phenotype of the ethanol drinker. In low-drinking females, as in males, KOR stimulation in the rostral NAc shell suppressed drinking. Consistent with this and with the effects of systemic KOR blockade on low-level continuous access drinking [37], we also found that antagonism of the KOR in the rostral NAc shell of female, low drinkers promoted ethanol drinking. In contrast to males, low-drinking females showed no effect of KOR stimulation in the caudal NAc shell on ethanol drinking, but high-drinking females responded to KOR stimulation in both the caudal and rostral shell with increased ethanol intake. In light of the lack of effect of KOR stimulation in the middle NAc shell on ethanol drinking in high drinkers, it may be that the middle NAc shell represents its own distinct zone, rather than being contiguous with the anterior and posterior subregions. Consistent with the effects of agonism but in contrast to effects in males, KOR antagonism in the caudal NAc shell of high-drinking females suppressed ethanol drinking. On the other hand, in the rostral NAc shell, antagonism of the KOR in high drinkers did not significantly affect ethanol intake. This is in contrast to studies performed in ethanol-dependent male rats that found systemic and local KOR blockade in the rostral NAc shell to suppress ethanol drinking [19, 27]. Nonetheless, as in males, our findings suggest that KOR activity is sufficient to alter ethanol drinking in a subregion-specific and level-dependent manner, but they suggest that it may only be necessary for effects on ethanol intake in the rostral NAc shell of low-drinking females and the caudal NAc shell of high-drinking females. Importantly, given that there are known sex-related but estrous independent effects of the KOR/dynorphin system on affective and motivated behavior [38, 39], and sex-dependent effects of KOR/dynorphin knockout on ethanol drinking [40, 41], we report that the effect of both KOR agonism and antagonism in the NAc shell on ethanol drinking is also influenced by subject sex.

As a possible explanation for these subregional effects, we found that there were subregional differences in baseline gene expression of KOR and dynorphin. That is, in ethanol naïve rats, both KOR and dynorphin mRNA levels were higher in the rostral compared to caudal NAc shell. While we tested this in female rats only, published evidence has shown that there are no baseline sex-related differences in KOR or dynorphin mRNA in the NAc [39]. We also found in the rostral NAc shell that gene expression of KOR, but not dynorphin, was upregulated in animals with a history of ethanol drinking. An upregulation of the KOR from high ethanol drinking may therefore underlie the change in KOR effects in the rostral NAc shell from inhibiting to stimulating ethanol drinking. Importantly, while real-time quantitative PCR may detect some mRNA in terminals, our results likely primarily capture differences in gene expression of KOR at the soma. It should be remembered that microinjections modulate the activity of both post- and pre-synaptic KORs, the latter of which are more numerous in the NAc shell [42]. While postsynaptic KORs are expressed on the medium spiny neurons of the NAc shell [43], presynaptic KORs are expressed on dopaminergic, GABA-ergic, and glutamatergic input from the ventral tegmental area, glutamatergic projections from the basolateral amygdala and prefrontal cortex, and serotonergic afferents from the dorsal raphe nucleus [1, 42–46]. Interestingly, dopamine activity in the rostral subregion has been found to prevent aversion, in contrast to dopamine in the caudal subregion, which induces it [47]. In rats, the rostral NAc shell also appears to be a unique site of convergence for hippocampal and basolateral amygdala projections [48], but KORs have been found to presynaptically inhibit the excitatory drive of basolateral amygdala but not hippocampal input [43]. There may, however, be important species-dependent differences, as the effects of the KOR on preference and aversion in mice appear to differ more along the dorsal-ventral rather than rostro-caudal axis [30]. Future studies should examine if there are differences in afferent projections and their KOR expression across rostro-caudal and dorsal-ventral subregions of the NAc shell in rats and mice.

## Conclusions

In summary, this study demonstrates that the activity of the KOR in the NAc shell can either inhibit or stimulate ethanol drinking, depending on the specific subregion targeted and the sex-related phenotype of the drinker. This effect is substance specific, and it may be explained by baseline and ethanol-dependent changes in the KOR/dynorphin system. These findings may inform the renewed clinical interest in the KOR as a pharmacological target for substance use disorders by highlighting sex and drinking phenotype as important factors in its effects.

### Supplementary information

Supplemental Information

Supplemental Statistics Table

## Data Availability

The datasets generated during and/or analyzed during the current study are available from the corresponding author on reasonable request.
