# Supplementary material for: Kappa-opioid receptor stimulation in the nucleus accumbens shell and ethanol drinking: Differential effects by rostro-caudal location and level of drinking
Source: Neuropsychopharmacology. 2024 Mar 25;49(10):1550–8. doi: 10.1038/s41386-024-01850-1 (PMC11319348; doi:10.1038/s41386-024-01850-1)
Supplement: Supplementary file 1 — Supplemental Information [file 41386_2024_1850_MOESM1_ESM.docx]

**Supplemental Information**

**Kappa-opioid receptor stimulation in the nucleus accumbens shell and ethanol drinking: Differential effects by rostro-caudal location and level of drinking**

Breanne E. Pirino, Ph.D.^1^, Annie Hawks, B.S.^1^, Brody A. Carpenter, M.S.^1^, Pelagia G. Candelas, M.S.^1^, Andrew T. Gargiulo, Ph.D.^1^, Genevieve R. Curtis, Ph.D.^1^, Anushree N. Karkhanis, Ph.D.^2^, Jessica R. Barson, Ph.D.^1^

^1^Department of Neurobiology and Anatomy, Drexel University College of Medicine, Philadelphia, P.A. 19129

^2^Department of Psychology, Binghamton University – SUNY, Binghamton, N.Y. 13902

**Supplemental Materials and Methods**

Ethanol & sucrose drinking (Experiments 1 - 4)

Rats were allowed to drink unsweetened 20% v/v ethanol or 2.5% w/v sucrose under the intermittent-access two-bottle-choice procedure (adapted from [1,2]), which consisted of three 24-hour sessions per week (Monday, Wednesday, and Friday), beginning 1.5 – 2 hours after dark onset, as described [3,4]. Animals had *ad libitum* access to water and chow. Ethanol or sucrose intake was measured for each access day and calculated as (weight ethanol or sucrose solution consumed (g) * (density ethanol or sucrose * concentration of ethanol or sucrose)) / rat body weight (kg). Animals were weighed on Tuesdays and Fridays. Preference for ethanol or sucrose was calculated as (volume of ethanol or sucrose solution consumed (ml)) / (volume of ethanol or sucrose solution consumed (ml) + volume of water consumed (ml)) * 100. Average ethanol or sucrose intake is reported and analyzed as grams of ethanol or sucrose per kilogram bodyweight. To calculate caloric (kcal) intake, ethanol intake was calculated as (density of ethanol * concentration of ethanol solution) * (weight of ethanol solution consumed (g)) * (calories per gram of ethanol), and for sucrose, it is calculated as (concentration of sucrose solution) * (weight of sucrose solution consumed (g)) * (calories per gram of sucrose). To determine blood ethanol concentration (BEC), trunk blood from a subset of rats from Experiments 1 – 3 (*N* = 23, 15 females and 8 males) was obtained at the termination of the experiments, 30 – 40 minutes after the start of daily ethanol access. Plasma was analyzed using an Analox AM1 Alcohol Analyzer (Lunenburg, MA, USA).

Drugs

The selective KOR agonist (±)-trans-U50,488 hydrochloride (U50,488) and the KOR antagonist nor-binaltorphimine dihydrochloride (nor-BNI) were acquired from Tocris (Minneapolis, MN, USA) and dissolved in 0.9% saline (Baxter International Inc., Deerfield, IL, USA) for microinjection at 0.8 nmol (0.34 μg) and 8.0 nmol (3.39 μg) per side for U50,488 [5] and 5.44 nmol (4 μg) for nor-BNI [6] in a volume of 0.3 µL. These doses have previously been found to alter behavior after injection into the NAc shell of male rats [5,6]. They have been demonstrated to be within the range of KOR selectivity [7-11]. We have previously demonstrated that injections at the volume used (0.3 μl) have a radial spread of approximately 0.5 mm and largely remain restricted within the NAc shell [3,12].

Microinjections (Experiments 1 - 3)

Rats were bilaterally cannulated using published methods [5]. Guide shafts (made from 21-gauge stainless steel tubing, Small Parts at Amazon.com, Seattle, WA, USA) were implanted perpendicularly, aimed at the rostral, middle, or caudal NAc shell (1.8 – 2.1, 1.4 – 1.6, or 0.8 – 1.1 mm anterior to Bregma, ±0.8 mm lateral to midline, 3.5 – 4.0 mm ventral to the level skull [13]. Bupivicaine (2 mg/kg s.c., Hospira Worldwide, Lake Forest, IL, USA) was injected into the scalp prior to incision, and buprenorphine hydrocholoride (0.03 mg/kg s.c., Reckitt & Colman Inc, Slough, UK) was administered for post-operative analgesia. To prevent occlusion, stylets (made from 26–gauge stainless steel tubing, Small Parts) were left in the guide shafts between injections. Rats were given at least one week to recover from surgery prior to the start of microinjections, and animals were handled daily during this time, with their stylet removed and replaced to acclimate them to the microinjection procedure.

For microinjections, rats were injected with freshly prepared U50,488, nor-BNI, or saline vehicle 1.5 – 2 hours (for U50,488 injections) or 4 hours (for nor-BNI) into the dark cycle, through microinjectors of 26–gauge stainless steel outside and fused-silica tubing inside (74 μm ID, 154 μm OD; Polymicro Technologies, Phoenix, AZ, USA) that extended 4.0 mm beyond the guide shafts to reach the NAc shell. A syringe pump (Harvard Apparatus, Holliston, MA, USA) delivered 0.3 μl of solution over 30 seconds, and the microinjector remained in place for an additional 30 – 60 seconds to allow for diffusion. Each side was injected sequentially, such that injection in one hemisphere for a single subject was immediately followed by injection into the other hemisphere. We have previously demonstrated that injections of methylene blue dye or a fluorescent dextran at the volume used (0.3 μl) have a radial spread of approximately 0.5 mm and largely remain restricted within the NAc shell [3,12]. With the NAc shell estimated to span almost 3 mm along the anterior-posterior axis of an adult rat, this suggests that our microinjections were largely restricted within each subregion, which each comprised roughly one-third of the brain region. The relatively smaller volume of the NAc shell in female than male rats means that there may have been relatively more overlap of drug diffusion across subregions in females; however, since the 3 mm estimation is based on a male rat weighing approximately 290 g, and our females weighed 259.09 ± 2.52 g (compared to males at 469.94 ±10.32 g) at the time of injection, it is reasonable to assume that the NAc shell of the rats included in our study were approximately 3 mm at the time of injection.

Quantitative real-time PCR (Experiment 4)

Female rats were sacrificed by rapid decapitation during the dark cycle at the time they would normally receive access to ethanol, and their rostral and caudal NAc shell was dissected out to examine mRNA levels of Oprk1 (KOR) and prodynorphin (DYN). Immediately after sacrifice, the brain was placed in a matrix slicing guide on ice, with the ventral surface facing up. Four coronal cuts were made, starting with the rostral optic chiasm (approx. Bregma −1.0 mm) [13]. The second and third cuts were 2.0 and then 1 mm rostral to this, yielding a slice (approx. Bregma 2.0 to 1.0 mm) for microdissection of the caudal NAc shell, and the fourth cut was 1.0 mm rostral to that, yielding a slice (approx. Bregma 3.0 to 2.0 mm) for the rostral NAc shell. Under a microscope, on a slide placed on a petri dish filled with ice, the rostral and caudal NAc shell were dissected bilaterally in a crescent moon shape, with the dorsal tip beginning at the lateral ventricle, the medial aspect at the semilunar nucleus, and the ventral edge along the ventral pallidum. These sections were then placed in RNAlater (Qiagen Inc., Valenia, CA, USA) and stored at −20 °C until extraction of RNA.

As previously described [14,15], total RNA from each brain section was purified using an RNeasy Mini Kit (Qiagen Inc.) and cDNA was reverse transcribed using SuperScript® VILO™ Master Mix (Invitrogen, Grand Island, NY, USA) in a SimpliAmp™ Thermal Cycler (Applied Biosystems, Waltham, MA, USA). To run the quantitative real-time PCR, the SYBR Green PCR core reagents kit (Applied Biosystems, Grand Island, NY, USA) was used in MicroAmp® Fast Optical 96-Well Reaction Plates (Applied Biosystems) on a StepOnePlus Real-Time PCR System (Applied Biosystems), under the conditions of 2 min at 50 °C (primer annealing), 10 min at 95 °C (polymerase activation and sequence extension), and 40 cycles of 15 s at 95 °C (denaturation) plus 1 min at 60 °C (annealing and extension). Each sample was run in triplicate. Target gene expression was quantified relative to cyclophilin-A using the relative quantification method (ΔΔC_T_). Primers were designed with the NCBI Primer design tool (<http://www.ncbi.nlm.nih.gov/tools/primer-blast/>) [16], and purchased from Invitrogen at ThermoFisher Scientific (Grand Island, NY, USA). Primer sequences were as follows: cyclophilin-A forward: 5′-GTGTTCTTCGACATCACGGCT-3′, reverse: 5′-CTGTCTTTGGAACTTTGTCTGCA-3′; dynorphin forward: 5′-CAGCGGACTGCCTGTCCTT-3′, reverse: 5′-TCAGGGTGAGAAAAGACCAAAAG-3′; KOR forward: 5′-CCAGTCTTGGAAGGCACAAGT-3′, reverse: 5′-CGACCTCCCTTCCCAAATCAG-3′. The target primers were used at a 100 nM concentration, while the housekeeping gene (cyclophilin) was used at a 200 nM concentration.

Histological analysis

Rats from Experiments 1 – 3 were sacrificed by rapid decapitation after brief CO2 exposure, and their brains were extracted. Microscopic examination of cresyl violet- (Nissl-) stained 30 μm coronal sections was used to ensure that injections were made into the medial NAc shell and determine the rostro-caudal subregion at which this occurred. Bregma +2.76 – +2.16 mm was considered rostral, Bregma +2.04 – +1.32 mm was considered middle, and Bregma +1.20 – +0.72 mm was considered caudal [13].

**Supplemental Results**

Ethanol & sucrose drinking

For average daily drinking, high drinkers consumed a significantly greater volume of both ethanol [*t*(75) = -11.645, *p* < 0.001] and sucrose [*t*(30) = -2.284, *p* = 0.030] than their low-drinking counterparts (Figure S1). Similarly, high drinkers also consumed significantly more calories from ethanol [*t*(76) = -8.318, *p* < 0.001] or sucrose [*t*(30) = -2.284, *p* = 0.030], respectively, than low drinkers (Figure S2).

**Figure S1.** Average volume of 24-hour ethanol and sucrose intake. **(A)** High **ethanol** drinkers consumed a significantly greater volume of ethanol than low drinkers. **(B)** High **sucrose** drinkers also consumed a significantly greater volume of sucrose solution than low drinkers. * *p* < 0.05 and *** *p* ≤ 0.001, two-tailed independent samples *t*-tests.


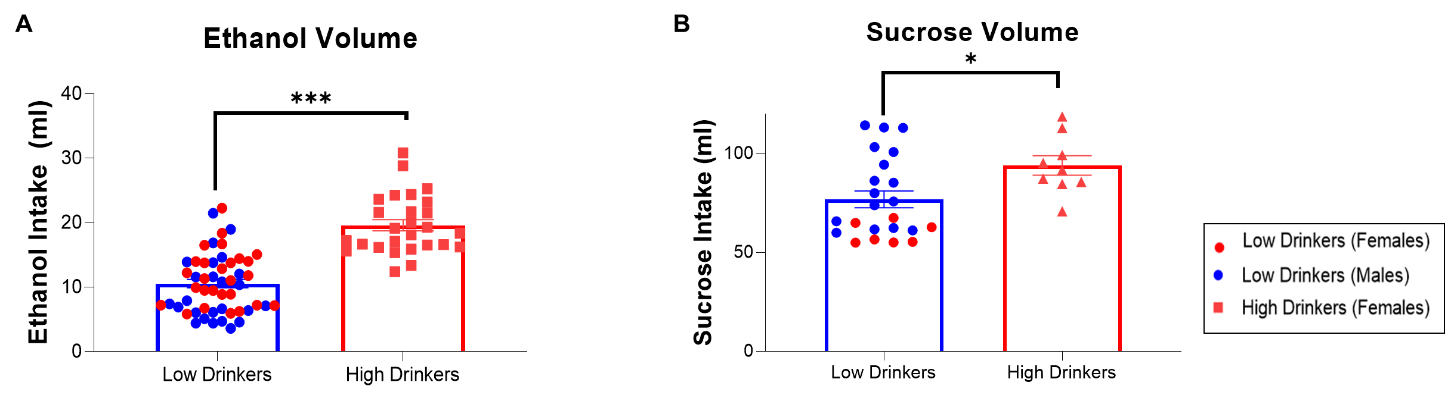


**Figure S2.** Average kcal from 24-hour ethanol and sucrose intake. **(A)** High **ethanol** drinkers consumed significantly more calories from ethanol than low drinkers. **(B)** High **sucrose** drinkers also consumed significantly more calories from sucrose than low drinkers. *** *p* < 0.05 and *p* ≤ 0.001, two-tailed independent samples *t*-tests.


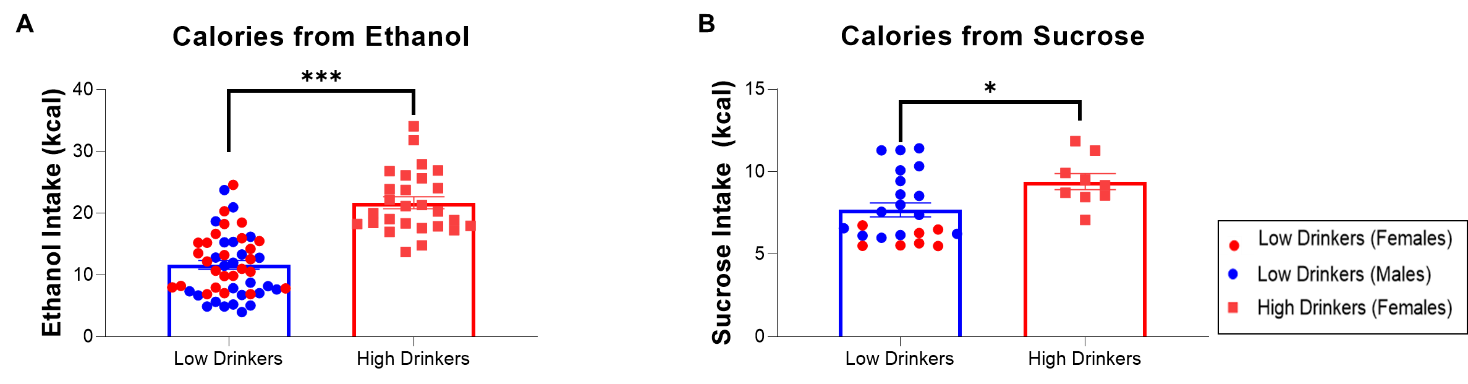


Histology

Histological examination confirmed that all injections were made into the medial NAc shell, with rostral NAc shell injections made between +2.76 and +2.16 mm from Bregma, middle NAc shell injections between +2.04 and +1.32 mm from Bregma, and caudal NAc shell injections between +1.20 and +0.72 mm from Bregma (Figure S3).


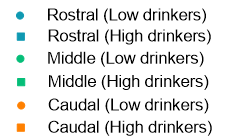


**A**

**B**

**C**

**D**


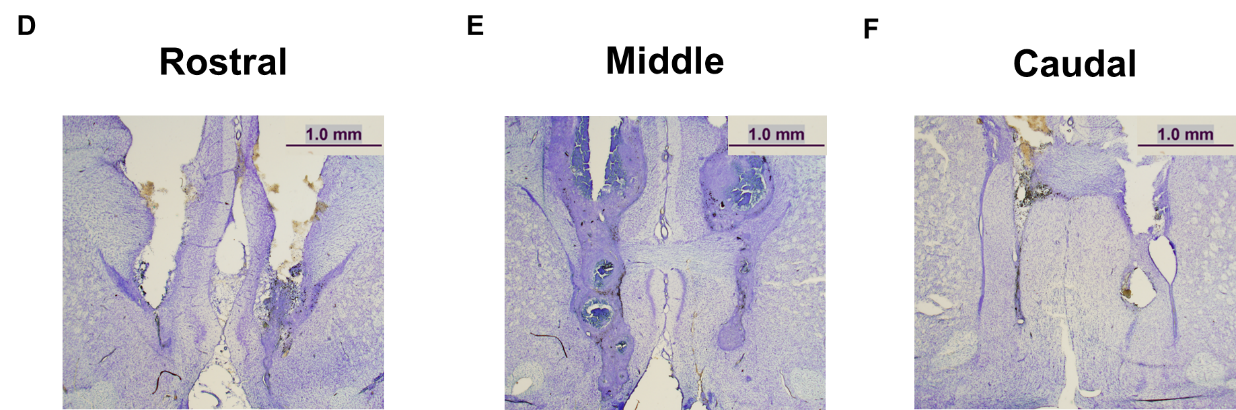

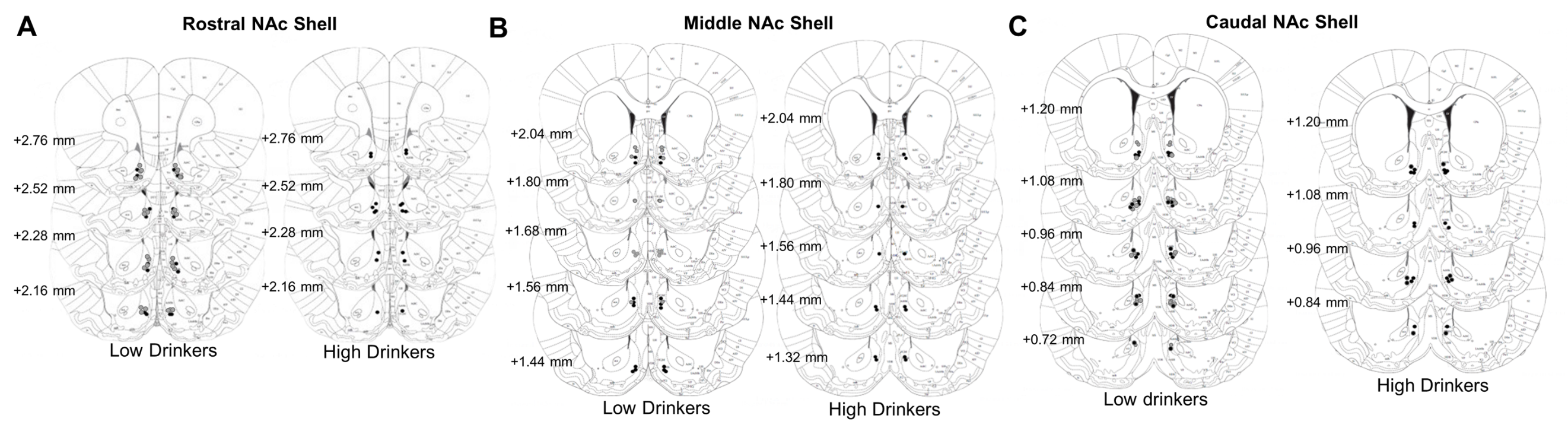


**Figure S3. (A – C)** Injection sites for all drugs injected into the **(A)** rostral, **(B)** middle, and **(C)** caudal NAc shell of high and low ethanol- and sucrose-drinking rats. Black circles indicate injection sites for female rats, and grey circles indicate injection sites for male rats [13]. Some circles represent the injection site for more than one animal. **(D – F)** Representative photomicrographs of injection sites in the **(A)** rostral, **(B)** middle, and **(C)** caudal NAc shell using tissue sliced coronally at 30 μm and stained with cresyl violet.


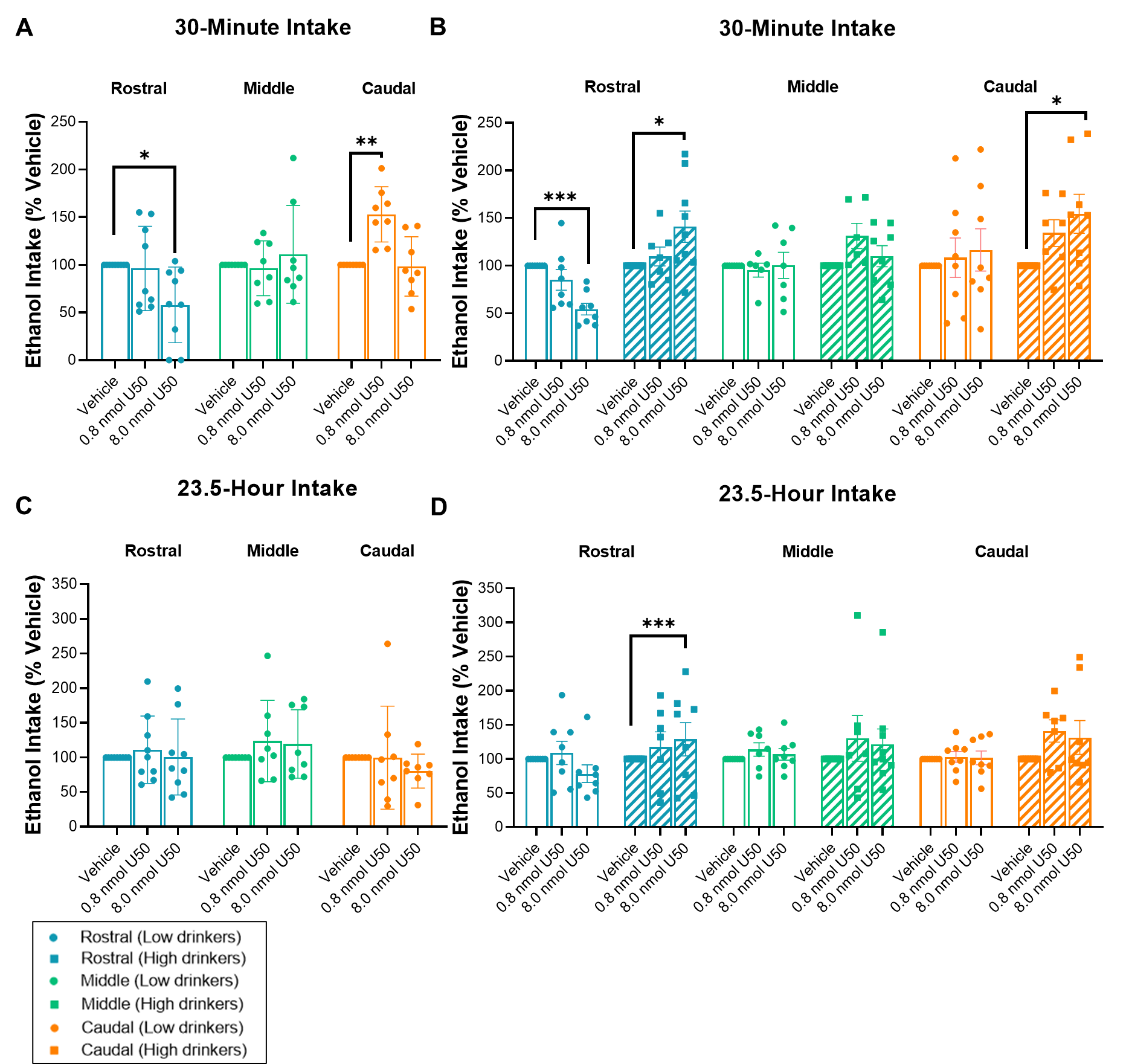


**Figure S4.** Effects of KOR stimulation on ethanol intake as a percentage of intake after vehicle injection. **(A)** In male rats, all of which are low drinkers, although there were no significant effects of U50,488 (U50) during the first 30 minutes of access in the *middle* NAc shell on ethanol drinking, 8.0 nmol U50 compared to saline vehicle in the *rostral* NAc shell significantly decreased ethanol drinking but 0.8 nmol U50 compared to saline vehicle in the *caudal* NAc shell significantly increased ethanol drinking. **(B)** For female rats, although there were no significant effects of U50 in the *middle* NAc shell on ethanol drinking during the first 30 minutes of access, 8.0 nmol U50 compared to saline vehicle injected into the rostral NAc shell significantly decreased ethanol drinking in low drinkers but significantly increased ethanol drinking in high drinkers, and the same dose of U50 also significantly increased ethanol drinking when injected into the *caudal* NAc shell of high drinkers. **(C)** There were no significant effects of U50 on ethanol drinking in male rats during the remaining 23.5 hours of access. (**D**) In high-drinking rats, all of which are female, 8.0 nmol U50 compared to saline vehicle injected into the *rostral* NAc shell significantly increased ethanol drinking during the remaining 23.5 hours of access. * *p* < 0.05, ** *p* < 0.01, and *** *p* ≤ 0 .001 vs. vehicle.

**Figure S5.** Effects of KOR stimulation on sucrose intake as a percentage of intake after vehicle injection. In male rats, all of which are low drinkers, stimulation of KORs with U50 had no effect on sucrose drinking **(A)** during the first 30 minutes of access or **(C)** during the remaining 23.5 hours of access when injected in either the rostral or caudal NAc shell. In female rats, stimulation of KORs with U50 had no effect on sucrose drinking **(B)** during the first 30 minutes of access or **(D)** during the remaining 23.5 hours of access on low drinkers or high drinkers when injected in the rostral NAc shell.


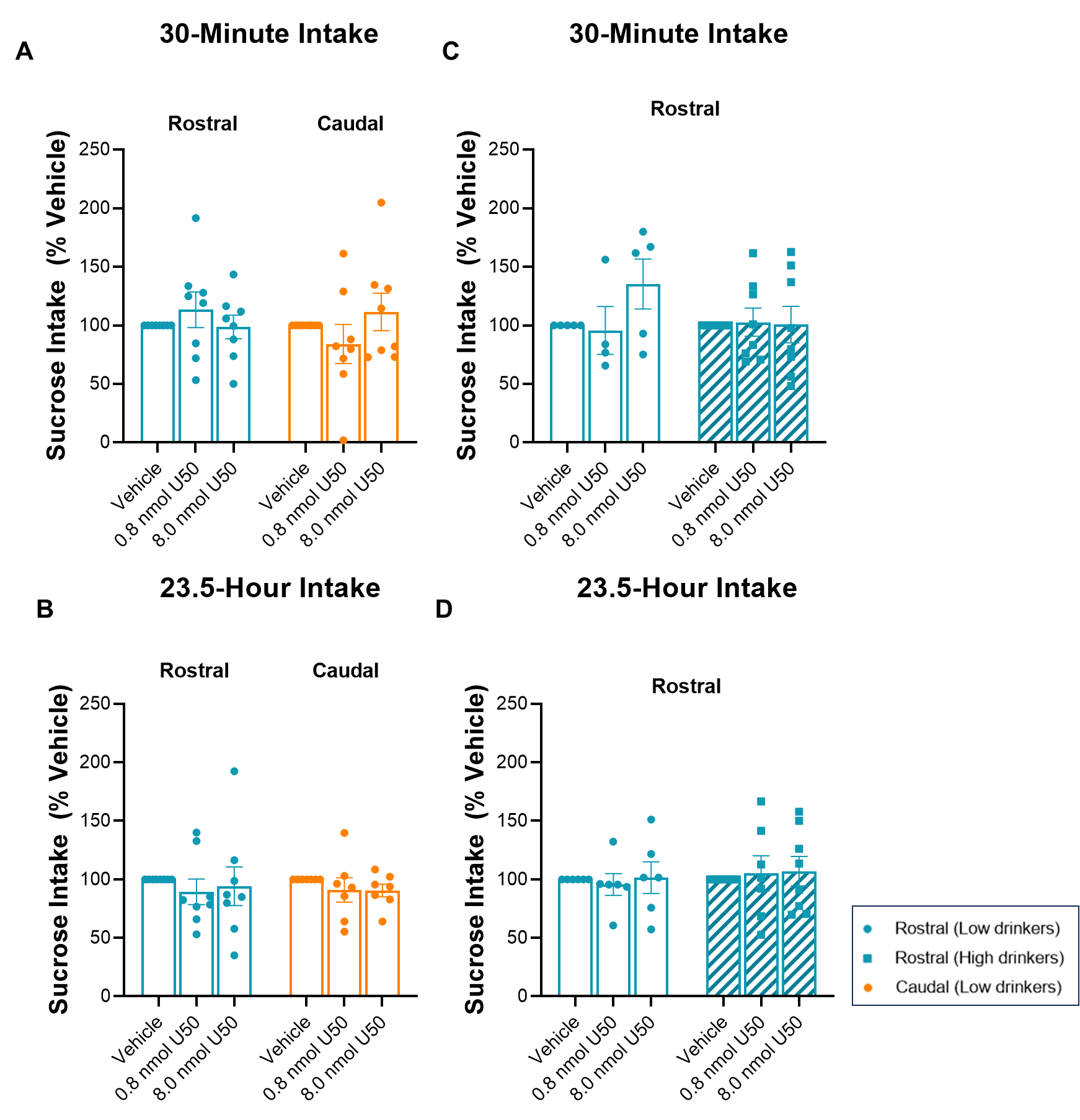


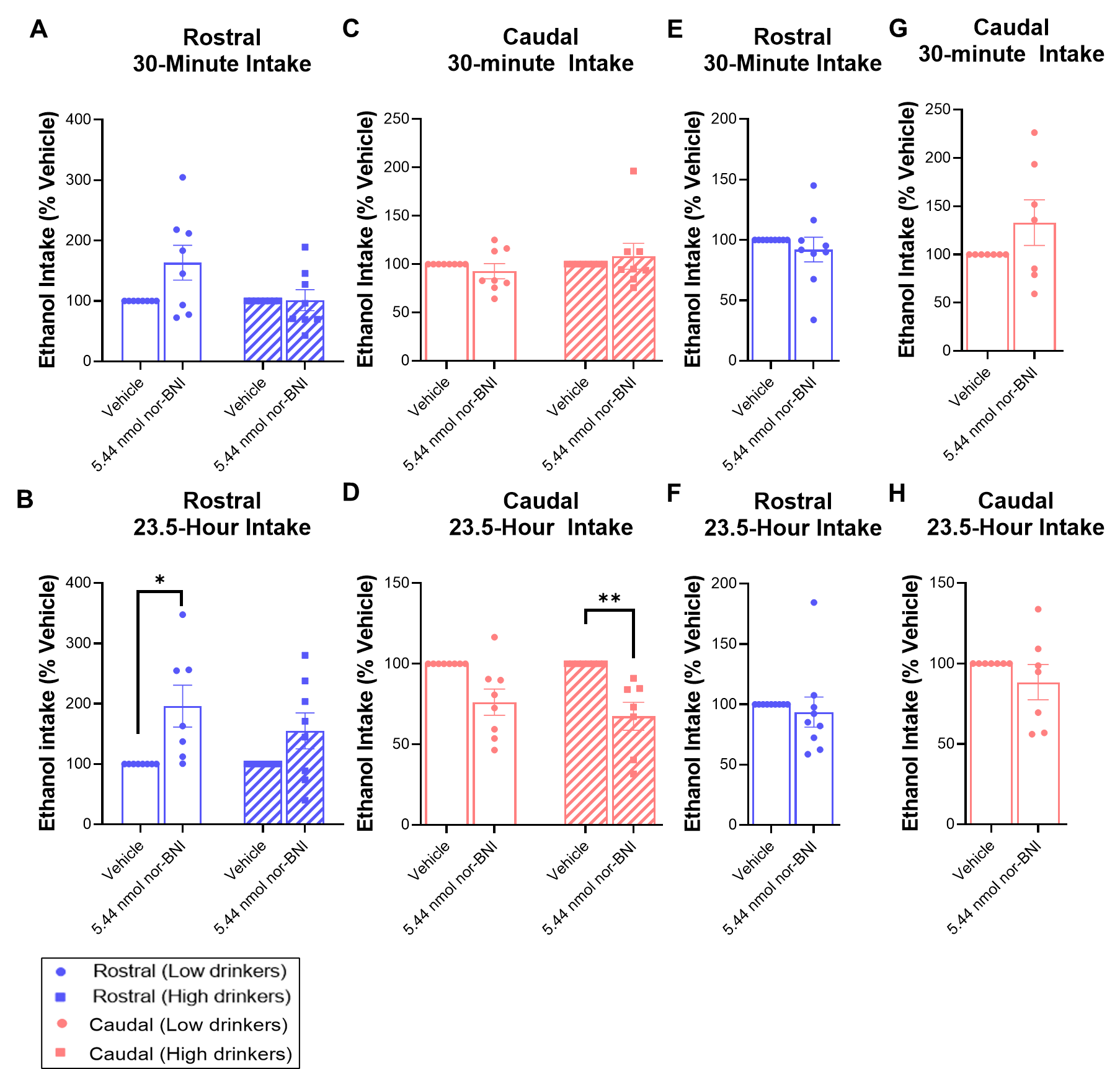


**Figure S6.** Effects of KOR blockade on ethanol intake as a percentage of intake after vehicle injection. **(A – D)** Effects of nor-BNI in female rats. In the *rostral* NAc shell, **(A)** nor-BNI had no effect on ethanol intake in females during the first 30 minutes of access, **(B)** but it promoted ethanol drinking in low drinkers during the remaining 23.5 hours of access. In the *caudal* NAc shell, **(C)** nor-BNI also had no effect on ethanol intake in females during the first 30 minutes of access, **(D)** but it suppressed drinking in high drinkers during the remaining 23.5 hours of access. **(E – H)** Effects of nor-BNI in male rats. In the *rostral* NAc shell, nor-BNI had no effect on ethanol drinking **(E)** during the first 30 minutes of access or **(F)** during the remaining 23.5 hours of access in male rats. In the *caudal* NAc shell, nor-BNI had no effect on ethanol drinking (**G**) during the first 30 minutes of access or **(H)** during the remaining 23.5 hours of access. * *p* < 0.05 and ** *p* < 0.01 vs. vehicle.

Food and water intake

**Experiment 1**: **Food and water intake**

**30-minute intake:** For simultaneously-available water intake, while a mixed ANOVA revealed a significant main effect of drug treatment [*F*(1.661, 93.007) = 4.060, *p* = 0.020] and a significant main effect of sex [*F*(1, 56) = 30.205, *p* < 0.001], there was no significant interaction between drug treatment, sex, and subregion [*F*(3.322, 93.007) = 1.452, *p* = 0.222] (data not shown). Sidak pairwise comparisons revealed that 8.0 nmol U50,488 compared to saline vehicle significantly decreased water intake (*p* = 0.017) and that male rats drank significantly more water than females (*p* < 0.001). For food intake, a mixed ANOVA revealed that there was no significant main effect of drug treatment [*F*(2, 114) = 0.367, *p* = 0.694], sex [F(1, 56) = 0.016, p = 0.900], or drinking phenotype [*F*(1, 57) = 0.132, *p* = 0.718] (data not shown). These results indicate that, although there are sex-related differences in baseline water drinking, KOR stimulation in the NAc shell does not alter food intake during the first 30 minutes of daily ethanol access.

**Remaining 23.5-hour intake**: For simultaneously-available water intake, although a mixed ANOVA revealed a significant main effect of sex [*F*(1, 60) = 19.179, *p* < 0.001], there was no significant main effect of drug treatment [*F*(2, 120) = 0.177, *p* = 0.889] or drinking phenotype [*F*(1, 60) = 2.729, *p* = 0.104] and no significant interaction between drug treatment, drinking phenotype, and subregion [*F*(4, 120) = 0.499, *p* = 0.737] (data not shown). Sidak pairwise comparisons revealed that male rats drank significantly more water than females (*p* < 0.001). For food intake, while a mixed ANOVA revealed a significant main effect of drinking phenotype [*F*(1, 58) = 6.513, *p* = 0.013] and sex [*F*(1, 58) = 68.353, *p* < 0.001], there was no significant main effect of drug treatment [*F*(2, 116) = 2.032, *p* = 0.136] and no significant interaction between drug treatment, drinking phenotype, and subregion [*F*(4, 116) = 0.437, *p* = 0.781] (data not shown). Sidak pairwise comparisons revealed that male rats consumed significantly more food than females (*p* < 0.001) and that low ethanol drinkers consumed significantly more food than high drinkers (*p* < 0.001). These findings indicate that, while there may be baseline sex-related differences in food and water intake and drinking phenotype-related differences in food intake, KOR stimulation in the NAc shell has no detectable effect on food or water intake after the first 30 minutes of daily ethanol access.

**Experiment 2**: **Food and water intake**

**30-minute intake:** For simultaneously-available water intake, a mixed ANOVA revealed that, while there was a significant main effect of sex [*F*(1, 20) = 13.549, *p* = 0.001] and a significant interaction between sex and drug treatment, there was no significant main effect of drug treatment [*F*(2, 40) = 0.453, *p* = 0.639], and Sidak pairwise comparisons revealed that, although male rats drank significantly more water than female rats (*p* < 0.001), there were no dose-dependent effects of U50,488 on water intake (*p* = 0.313 – 0.999) (data not shown). For food intake, a mixed ANOVA revealed that there was no significant main effect of drug treatment [*F*(2, 40) = 0.272, *p* = 0.763], sex [*F*(1, 20) = 0.856, p = 0.784], or drinking phenotype [*F*(1, 20) = 0.898, *p* = 0.355] (data not shown). Thus, despite baseline sex-related differences in water intake, KOR stimulation in the NAc shell has no effect on food or water intake during the first 30 minutes of sucrose access.

**Remaining 23.5-hour intake**: For simultaneously-available water intake, a mixed ANOVA revealed that there was no main effect of drug treatment [*F*(2, 36) = 1.119, *p* = 0.338], sex [*F*(1, 18) = 2.359, *p* = 0.142], or drinking phenotype [*F*(1, 18) = 0.395, *p* = 0.538] (data not shown). For food intake, although a mixed ANOVA revealed a significant main effect of sex [*F*(1, 22) = 41.491, *p* < 0.001], there was no significant main effect of drug treatment [*F*(2, 44) = 0.770, *p* = 0.469] or drinking phenotype [*F*(1, 22) = 0.479, *p* = 0.496] (data not shown). Sidak pairwise comparisons revealed that male rats consumed significantly more chow than female rats (*p* < 0.001). These data suggest that, while there are some sex-related differences in sucrose drinking after the first 30 minutes of access, KOR activation in the NAc shell does not alter sucrose intake.

**Experiment 3**: **Food and water intake**

**30-minute intake:** For simultaneously-available water intake, a mixed ANOVA revealed that there was no significant main effect of drug treatment [*F*(1, 15) = 0.094, *p* = 0.764] or drinking phenotype [*F*(1, 15) = 0.281, *p* = 0.603] (data not shown). For food intake, a mixed ANOVA revealed that there was also no significant main effect of drug treatment [*F*(1, 15) = 0.079, *p* = 0.782] or drinking phenotype [*F*(1, 15) = 1.504, *p* = 0.204] (data not shown). Thus, KOR blockade did not affect food or water intake during the first 30 minutes of ethanol access.

**Remaining 23.5-hour intake:** For simultaneously-available water intake, a mixed ANOVA revealed that there was no significant main effect of drug treatment [*F*(1, 15) = 2.121, *p* = 0.166] or drinking phenotype [*F*(1, 15) = 0.307, *p* = 0.588] (data not shown). Similarly, for food intake, a mixed ANOVA revealed that there was no significant main effect of drug treatment [*F*(1, 15) = 0.422, *p* = 0.526] or drinking phenotype [*F*(1, 15) = 0.052, *p* = 0.823] (data not shown).These findings suggest that there was no effect of KOR blockade on food or water intake after the first 30 minutes of ethanol access.

**References**

1 Wise RA. Voluntary ethanol intake in rats following exposure to ethanol on various schedules. Psychopharmacologia. 1973;29(3):203-10.

2 Simms JA, Steensland P, Medina B, Abernathy KE, Chandler LJ, Wise R, et al. Intermittent access to 20% ethanol induces high ethanol consumption in Long-Evans and Wistar rats. Alcohol Clin Exp Res. 2008;32(10):1816-23.

3 Barson JR, Ho HT, Leibowitz SF. Anterior thalamic paraventricular nucleus is involved in intermittent access ethanol drinking: role of orexin receptor 2. Addict Biol. 2015;20(3):469-81.

4 Pirino BE, Martin CR, Carpenter BA, Curtis GR, Curran-Alfaro CM, Samels SB, et al. Sex-related differences in pattern of ethanol drinking under the intermittent-access model and its impact on exploratory and anxiety-like behavior in Long-Evans rats. Alcohol Clin Exp Res. 2022;46(7):1282-93.

5 Pirino BE, Spodnick MB, Gargiulo AT, Curtis GR, Barson JR, Karkhanis AN. Kappa-opioid receptor-dependent changes in dopamine and anxiety-like or approach-avoidance behavior occur differentially across the nucleus accumbens shell rostro-caudal axis. Neuropharmacology. 2020;181:108341.

6 Nealey KA, Smith AW, Davis SM, Smith DG, Walker BM. kappa-opioid receptors are implicated in the increased potency of intra-accumbens nalmefene in ethanol-dependent rats. Neuropharmacology. 2011;61(1-2):35-42.

7 Jenck F, Bozarth M, Wise RA. Contraversive circling induced by ventral tegmental microinjections of moderate doses of morphine and [D-Pen2, D-Pen5]enkephalin. Brain Res. 1988;450(1-2):382-6.

8 Massaly N, Copits BA, Wilson-Poe AR, Hipolito L, Markovic T, Yoon HJ, et al. Pain-Induced Negative Affect Is Mediated via Recruitment of The Nucleus Accumbens Kappa Opioid System. Neuron. 2019;102(3):564-73 e6.

9 Takemori AE, Ho BY, Naeseth JS, Portoghese PS. Nor-binaltorphimine, a highly selective kappa-opioid antagonist in analgesic and receptor binding assays. J Pharmacol Exp Ther. 1988;246(1):255-8.

10 Tortella FC, Echevarria E, Lipkowski AW, Takemori AE, Portoghese PS, Holaday JW. Selective kappa antagonist properties of nor-binaltorphimine in the rat MES seizure model. Life Sci. 1989;44(10):661-5.

11 Spanagel R, Almeida OF, Shippenberg TS. Evidence that nor-binaltorphimine can function as an antagonist at multiple opioid receptor subtypes. Eur J Pharmacol. 1994;264(2):157-62.

12 Gargiulo AT, Pirino BE, Curtis GR, Barson JR. Effects of pituitary adenylate cyclase-activating polypeptide isoforms in nucleus accumbens subregions on ethanol drinking. Addict Biol. 2021;26(3):e12972.

13 Paxinos G, Watson C. Paxino's and Watson's The rat brain in stereotaxic coordinates*.* Seventh edition. ed. Elsevier/AP, Academic Press is an imprint of Elsevier: Amsterdam ; Boston; 2014.

14 Gargiulo AT, Badve PS, Curtis GR, Prino BE, Barson JR. Inactivation of the thalamic paraventricular nucleus promotes place preference and sucrose seeking in male rats. Psychopharmacology (Berl). 2022;239(8):2659-71.

15 Pandey S, Badve PS, Curtis GR, Leibowitz SF, Barson JR. Neurotensin in the posterior thalamic paraventricular nucleus: inhibitor of pharmacologically relevant ethanol drinking. Addict Biol. 2019;24(1):3-16.

16 Ye J, Coulouris G, Zaretskaya I, Cutcutache I, Rozen S, Madden TL. Primer-BLAST: a tool to design target-specific primers for polymerase chain reaction. BMC Bioinformatics. 2012;13:134.
